# Preventing Multimorbidity with Lifestyle Interventions in Sub-Saharan Africa: A New Challenge for Public Health in Low and Middle-Income Countries

**DOI:** 10.3390/ijerph182312449

**Published:** 2021-11-26

**Authors:** Ahmad Alkhatib, Lawrence Achilles Nnyanzi, Brian Mujuni, Geofrey Amanya, Charles Ibingira

**Affiliations:** 1 School of Health and Life Sciences, Teesside University Tees Valley, Middlesbrough TS1 3BX, UK; l.nnyanzi@tees.ac.uk; 2 College of Health Sciences, Makerere University, Kampala P.O. Box 7072, Uganda; bmujuni7@gmail.com (B.M.); cibingira@gmail.com (C.I.); 3 Ministry of Health, 6 Lourdel Road, Nakasero, Kampala P.O. Box 7272, Uganda; geofreyamanya@gmail.com

**Keywords:** multimorbidity, non-communicable diseases, lifestyle prevention, public health, low and middle-income countries

## Abstract

Objectives: Low and Middle-Income Countries are experiencing a fast-paced epidemiological rise in clusters of non-communicable diseases such as diabetes and cardiovascular disease, forming an imminent rise in multimorbidity. However, preventing multimorbidity has received little attention in LMICs, especially in Sub-Saharan African Countries. Methods: Narrative review which scoped the most recent evidence in LMICs about multimorbidity determinants and appropriated them for potential multimorbidity prevention strategies. Results: MMD in LMICs is affected by several determinants including increased age, female sex, environment, lower socio-economic status, obesity, and lifestyle behaviours, especially poor nutrition, and physical inactivity. Multimorbidity public health interventions in LMICs, especially in Sub-Saharan Africa are currently impeded by local and regional economic disparity, underdeveloped healthcare systems, and concurrent prevalence of communicable and non-communicable diseases. However, lifestyle interventions that are targeted towards preventing highly prevalent multimorbidity clusters, especially hypertension, diabetes, and cardiovascular disease, can provide early prevention of multimorbidity, especially within Sub-Saharan African countries with emerging economies and socio-economic disparity. Conclusion: Future public health initiatives should consider targeted lifestyle interventions and appropriate policies and guidelines in preventing multimorbidity in LMICs.

## 1. Background and Objectives

Global mortality among adults is primarily caused by non-communicable diseases (NCDs) of cardiovascular disease (CVD), cancer, respiratory disease, and diabetes, with related annual mortality records showing 17.9, 9, 3.9 and 1.6 million respectively for each disease [1]. These NCDs are known to be preventable through addressing their modifiable risk factors. Unfortunately, of those figures, 85% (15 million) NCDs-related mortalities are in low and middle-income countries (LMICs) [1]. However, recent reports have shown LMICs suffer a more rapid increase in mortality or disability burdens attributable to multiple NCDs (e.g., cancer, diabetes chronic respiratory disease, mental and substance use disorders) from 45% between 1990 and 2010, to 67% between 2010 and 2017, which is a higher rise rate than NCDs in high-income countries (HICs) [2,3,4]. The majority of LMICs are based in the Sub-Saharan African region, which represents countries with a similar disease profile characterised by emerging and re-emerging infectious communicable diseases (CDs) such as malaria, HIV/AIDS and tuberculosis, and under-resourced health systems. The recent epidemiological transition from CDs to multiple NCDs in Sub-Saharan Africa signifies a higher risk of mortality and disease-related disability than other global regions over the coming decades [5,6]. Therefore, there is a strong case for early prevention of NCDs multimorbidity in LMICs, especially Sub-Saharan African countries.

Multimorbidity is defined as a phenomenon where two or more chronic conditions exist concurrently in the same individual, such as diabetes, respiratory disease, cancer, and CVD [7,8]. Multimorbidity implies any co-occurrence of medical conditions, which has long been distinguished from comorbidity, which entails a burden of illness co-existing with a particular disease [9]. Multimorbidity is often referred to in the context of age-related NCDs, or a concomitant disability related to NCD burdens within an ageing population, which persist over time causing physical and mental health decline and premature mortality [5,10]. However, multimorbidity is present in all age groups and in both genders. Systematic associations between chronic diseases, which tend to cluster in multimorbidity patterns, have been identified even in the paediatric population [7], which necessitates an implementation of preventive strategies, especially in younger adults.

Associations between chronic diseases are often disparate, which makes it difficult to devise effective care models and interventions. For example, circulatory diseases were associated with respiratory disorders, chronic musculoskeletal diseases with depression and anxiety, and a consistent pattern of metabolic syndrome (hypertension, diabetes, obesity, and dyslipidaemia), among others [11]. Considering the complex systematic associations between chronic diseases, various multimorbidity patterns have been identified in the general population and many researchers call for person-centered approaches and effective multimorbidity care models [11,12].

Cohort studies have often focused on HICs, whilst demonstrating over 66% multimorbidity prevalence in older adults (>55 years old), with increased hospital visit burdens and healthcare costs [13,14,15]. However, within LMICs, especially Sub-Saharan Africa, poverty and CDs prevalence are likely to add to multimorbidity burdens. For example, poverty indicated by a 0.5% decrease in economic growth has been associated with 10% increase in multiple NCDs [16] and is coupled with little knowledge about multimorbidity models of care and prevention in LMICs [17].

Recent limited data from the Sub-Saharan African region shows an emerging trend in the prevalence of multimorbidity, which may be preventable if addressed early. For example, Uganda reported 36.9 and 26.4% prevalence of pre-hypertension and hypertension respectively, concurrent with obesity and to a less extent with impaired fasting glucose (2%) and diabetes (1.2%), which were explained by modifiable factors of excess body weight (body mass index, BMI ≥ 25 kg m^−2^) and undiagnosed and uncontrolled blood pressure [18]. Such high prevalence was close to a 31% median (range of 17–40%) reported across 31 Sub-Saharan African LMICs using the same WHO STEP-wise NCDs survey approach (sociodemographic questionnaires, physical examination, and biochemical assessments) [19]. The increased multimorbidity prevalence (e.g., CVD, diabetes, chronic respiratory disease and cancer) in LMICs is likely to be explained by modifiable lifestyle risk factors, including unhealthy diets, physical inactivity, obesity, tobacco and alcohol use [16]. However, initial multimorbidity preventative attempts in LMICs were faced by increased healthcare costs, which could further impede poverty-reduction strategies and exacerbate a vicious cycle of poverty, ill-health, and underdevelopment [20]. However, future interventions in Sub-Saharan Africa could address a cluster of emerging NCDs forming multimorbidity (e.g., diabetes, hypertension and CVD) through lifestyle interventions, especially when such approaches are personalised towards local needs. This narrative review summarises key epidemiological multimorbidity determinants and highlights specific public health challenges related to multimorbidity prevention strategies, especially through lifestyle in LMICs placing emphasis on Sub-Saharan Africa, and its future regionally-relevant health policies.

## 2. Methodology

This narrative review focused on preventing MMD through mapping its determinants with potential lifestyle prevention approaches in Sub-Saharan African LMICs. A systematic search was undertaken to obtain primary studies and other sources relevant to the review topic. The keywords and synonyms used in the search were derived from Medical Subject Headings (MeSH) and a scoping search. These terms were combined with Boolean operators ‘OR’ and ‘AND’ to ensure balanced sensitivity and specificity in the search respectively. These terms included: multimorbid* OR “non-communicable diseas*” OR “communicable diseas*” OR “multiple chronic conditions” OR MMD* OR “multiple diseases” AND “lifestyle prevention” OR “lifestyle medicin* OR diet* OR “physical active*” AND LMIC* OR “low and middle income countr*” OR Sub Sahara* Africa OR SSA*. Six (6) electronic databases namely PubMed, EMBASE, EBSCO, CINAHL, Cochrane Database and Google Scholar were searched between May to June 2020 and a repeat search was conducted in July 2021. In searching the databases, the recommended unique syntax, and symbols (truncations or wildcards) were used. A supplementary search for grey literature and studies not indexed in selected databases was conducted on google. The first 15 pages of results were retained and examined for relevant articles. Hand searching of the reference list for all included studies was conducted to identify any related evidence sources. No time or language restrictions were applied in the course of the systematic search to allow for robustness. All studies obtained from database and grey literature search were transferred to Endnote 20 for storage, duplicate removal, and study selection based on predetermined PIO (population, intervention, outcome) eligibility criteria including: (1) study population being participants in an LMIC, (2) intervention being life-style prevention of disease and (3) outcome being multi-morbidity. A total of 6.058 citations were retrieved from the systematic search. After duplicate removal, 1.065 citations were screened through a two-stage selection process recommended by the Centre for Reviews and Dissemination. First, the titles and abstracts were screened against the eligibility criteria to identify relevant papers and then selected papers were screened on full-text against the same criteria. Two independent reviewers conducted the two-stage selection to allow for reliability and avoidance of reviewer fatigue. Studies that did not meet the inclusion criteria were excluded and reasons for such exclusion were stated (see Prisma flow diagram). Discrepancies between reviewers were discussed and resolved through a consensus.

**Figure 1:**
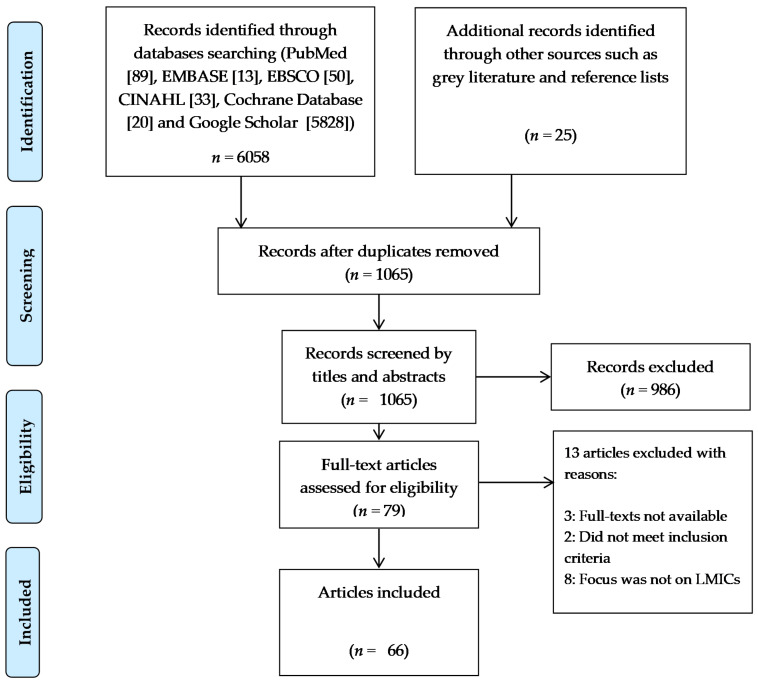
PRISMA Flow Diagram.

The Preferred Reporting Items for Systematic Reviews and Meta-Analyses (PRISMA) flow diagram was used in reporting study selection processes (Figure 1). The quality of all the primary studies included in this systematic review was critically appraised by the reviewers however no study was eliminated due to its methodological quality as the goal was to scope all relevant literature on the review topic. Quality of the study was commented on in the write-up of the various sections. Data from included studies were extracted, analysed, and presented as textual narratives in combination with tables highlighting relevant outcomes.

## 3. Determinants of Multimorbidity and Its Increased Burden in LMICs

We have seen an increased focus on multimorbidity prevalence and associated determinants in LMICs. Table 1 summarizes key multimorbidity determinants across representative LMICs from different global regions and population groups (e.g., Sub-Saharan Africa, Asian region) based on cross-sectional, national surveys and systematic reviews (Table 1).

The summarized evidence suggests a higher multimorbidity prevalence amongst populations within LMICs compared with HICs, which could be explained by modifiable lifestyle factors (Table 1). Below is a critical summary of the main multimorbidity determinants in LMICs.

### 3.1. Age

Although multimorbidity is present in all age groups, older age is a known risk factor of multimorbidity and associated disabilities. A worldwide increase in life expectancy above 75 years of age was concurrent with an increased prevalence of multimorbidity’s cluster of diabetes, hypertension, CVD, and associated healthcare cost [20,21,22,23,24]. A recent estimate of multimorbidity prevalence amongst older individuals in a HIC showed 67.5% [22]. Systematic reviews showed age to be positively associated with frequent patterns of two or more metabolic, cardiovascular, and physical chronic conditions in global [23], and European cohorts [24]. The latter analysis including over 70 million patients within primary care settings between 1961 and 2013 across 12 countries indicated disparate age-dependent prevalence, where multimorbidity was highly prevalent in older adults (95.1% in those ≥65 years old) than in younger adults (12.9% in those ≥18 years old). However, it was apparent in those studies that, together with older age, multimorbidity is also determined by socio-economic status (e.g., higher multimorbidity prevalence associated with low economic status), alongside sex (female sex), and mental health issues [23,24]. Furthermore, poorer LMICs have reported a more rapid age-dependent rise in multimorbidity, especially within Sub-Saharan African countries experiencing a simultaneous rapid economical and age growth [21,22]. Thus, prevention should target multimorbidity in LMICs regions with such rapid growth.

There is already evidence of reported age-dependent economic disparity between LMICs and HICs. A cross-sectional analysis within 28 countries showed a low multimorbidity prevalence of 7.8% when all ages were combined in LMICs compared with 66.5% in HICs, but this rose considerably with age (to around 21%), and such prevalence was associated (non-linear relationship) with country’s GDP [25]. An economic disparity coupled with an ageing population in HICs vs. LMICs may reflect different multimorbidity burdens. For example, an analysis of a single LMIC (Bangladesh) showed a significantly higher multimorbidity prevalence of 56.4% amongst older (>65 years old) individuals, and a higher prevalence in females (64.18%) than males (54.17%) [26]. The most prevalent conditions were hypertension (33.0%), diabetes (27.6%), ischemic heart disease (12.0%), and chronic obstructive pulmonary disease (9%) [26]. However, this remains a lower prevalence in comparison with HICs, which showed a prevalence of hypertension, dyslipidemia, diabetes, pain disorders, depression, heart failure, cancer, and dementia among the older adults of 60.6%, 51.2%, 25.2%, 34.0%, 12.0%, 14.0%, 8.6%, and 8.4%, respectively [27]. A more recent comparison of pooled multimorbidity prevalence showed 37.9% (95% CI: 32.5–43.4%) of multimorbidity in HICs compared with 29.7% (26.4–33.0%) in LMICs [28]. Such age-dependent discrepancy in multimorbidity burdens between HICs and LMICs may be explained by differences in age expectancy since a significant portion of people affected by NCDs in low- and middle-income economies die before reaching the age of 70 years old [29].

### 3.2. Hypertension

There is a higher prevalence of hypertension among adults in LMICs (31.5%, 1.04 billion people) than in HICs (28.5%, 349 million people) [30]. Heterogeneity of prevalence was explained by variations in the levels of risk factors for hypertension, such as high sodium intake, low potassium intake, obesity, alcohol consumption, physical inactivity and unhealthy diet [30]. Hypertension is a risk factor for ischemic heart disease, which is responsible for 71% of global mortalities [31].

Limited number of studies within LMICs have often reported obesity concurrently with hypertension [18,32], and to a less extent impaired glucose tolerance and diabetes [18,19,33]. One of the most common and consistent multimorbidity patterns (in both genders and in many age groups) is the metabolic one, which includes hypertension, diabetes mellitus, dyslipidaemia, and obesity [11]. For diabetes (fasting blood glucose ≥ 6.1 mmol l^−1^), a wider range of heterogeneity was reported with a prevalence range from 3% in Togo and Benin to 23% in Niger, and a median of 8% across the Sub-Saharan region, which was also explained by lifestyle factors (e.g., dietary intake habits, physical activity) [20]. This suggests that lifestyle interventions focused on a multimorbidity cluster involving hypertension could be useful also for preventing diabetes in LMICs. However, most Sub-Saharan Africa LMICs, with recent affliction of hypertension also suffer from inadequate diagnosis, awareness, or treatment strategies [32]. In one study, an average of only 27% of participants knew their hypertensive status and 18% were being treated for hypertension [32]. An analysis of the “Demographic and Health Survey (DHS)” data of 33 countries in the Sub-Saharan African region showed a particularly higher hypertension prevalence in women of reproductive age [33]. This analysis also reported a local disparity, where hypertension was highest among women in Lesotho with (17.3%) and was lowest among women in Burundi (1.0%). Hypertension co-existed with other contrasting health conditions, such as anaemia (60%) which was among Sub-Saharan African women, especially in Gabon, and obesity, especially in Lesotho (19.9%), Gabon (18.9%) and Ghana (15.6%), but not in Madagascar (1.1%) [33]. Interestingly, this analysis also found that the behavioural or modifiable risk factors of hypertension and obesity were; smoking, fruits, vegetables and alcohol consumption, while non-behavioural primarily non-modifiable significant factors included age, residence, religion, education, wealth index, marital status, employment, and number of children ever born. This suggests the importance of promoting locally-relevant lifestyle behavioural interventions, additionally to screening and treatment to mitigate hypertension and associated multimorbidity clusters in LMICs.

### 3.3. Environmental Factors and Rural Living

#### 3.3.1. Environmental Factors

Long term negative impact of environmental factors on health outcomes has particularly focused on air pollution associations with the development of chronic asthma, pulmonary insufficiency, CVD, cancer, and diabetes [34,35]. A cross-sectional analysis of 6.392 participants of the Swiss Cohort Study on Air Pollution, Lung and Heart Diseases in adults found an association between long term air pollution exposure and diabetes mellitus [36]. LMICs could experience a greater negative health impact of such environmental factors because of overpopulation and rapid urbanisation coupled with developing industrialisation [37]. For example, some LMICs rely on wood fuel, which still forms a significant part of fuel supply due to poverty, which exposes populations to poorer air quality and increased risk of multimorbidity [38]. There is also evidence of an association between long term indoor exposure to air pollution and cancer in LMICs [39], and CVD and related mortalities [40]. For example, a cross-sectional study in Kenya involving 5% sample of health visitors at a clinic at Trnava University located in Mukuru slum in Nairobi, reported that environmental conditions were the main determinant of health complaints amongst those visitors [41]. Conditions including asthma, pharyngitis and respiratory infections were concurrent with virosis, gastritis and enteritis among both males and females [41]. Thus, poor environmental factors, especially air pollution remain a major predictor of multimorbidity in LMICs, and necessitates an evidence-based approach when designing lifestyle interventions.

#### 3.3.2. Rural Living

Recent evidence suggested urban-rural differences in multimorbidity in China and Korea, where 24% of the study population had multimorbidity, with higher proportions in rural areas whilst in China, 31% of the sample had multimorbidity, with significantly higher proportions in the cities [42]. The latter’s multivariate analysis also showed that rurality was associated with a lower risk of multimorbidity in both China and Korea, but with a socioeconomic disparity around urban areas in Korea [42]. However, contradictory findings in HICs in southern Europe, reported that those living in a rural area had a higher prevalence of three or more acute diseases, compared with patients with no acute diseases [43]. In an LMIC such as Uganda, key risk factors for NCDs were assessed using a nationally representative survey involving 3987 participants. It was concluded that the relative risk of living in a rural area significantly increased the risk of having one or two risk factors for multiple NCDs, which was 1.6 fold higher compared with living in the urban areas, though it is not clear whether such risks explain any multimorbidity (e.g., hypertension, obesity and diabetes) prevalence surveyed [44]. Limited or equivocal evidence makes it difficult to conclude whether rurality is a cause or just a confounder for multimorbidity in LMICs, which requires further research.

### 3.4. Lifestyle Factors

Lifestyle is a known determinant of multimorbidity in developed HICs. Modifiable lifestyle risk factors such as smoking habits, alcohol consumption, fruit and vegetable consumption, physical activity or their risk factors such as BMI (obesity) have been all associetd with multimorbidity [16,45]. Health related quality of life is another indicator of multimorbidity, which encompass measures of lifestyle factors, and was found to be negatively associated with multimorbidity among 18,137 adults in China [46]. LMICs, which suffer from health disparities related to the effects of lifestyle have received limited research attention. For example, an Egyptian cohort showed that lifestyle factors (BMI, fruit and vegetable intake) were major multimorbidity determinants, and the cumulative multimorbidity was related to region, residence, type of residence and land tenure system [47]. A systematic review of 24 interventions in LMICs found that none of those studies reported on the global NCD indicators relating to the intake of saturated fat, salt, physical inactivity, alcohol or tobacco use [16]. Instead, the majority reported on undernutrition (via dietary diversity, micronutrient deficiencies and underweight), fortified crop intake, mitigating water contamination and one on reduced physical exertion from manual labor [16]. Limited evidence suggests that lifestyle interventions should address both health and economic disparity to have great potential to impact NCDs prevalence and risk in LMICs.

No data is currently available on how lifestyle modifiable factors could be targeted to prevent multimorbidity within specific LMICs, despite recently suggested protocols about raising awareness of lifestyle prevention of obesity, physical inactivity, smoking, inappropriate use of alcohol and psychosocial factors (e.g., negative life events, social networks, mental health problems) in LMICs primary healthcare systems [17].

Lifestyle interventions as a multimorbidity prevention strategy need to consider health disparity within LMICs which suffer from the concurrent prevalence of NCDs and CDs. Analysis of cross-sectional data from 3889 people enrolled in the Health and Ageing in Africa longitudinal study in South Africa found that the negative multimorbidity association with age, wealth, and lifestyle factors of physical functioning and well-being was due to different epidemiological CDs profile in subgroups [48]. This analysis, which reported on both CDs (HIV, anaemia) and NCDs (cardiometabolic conditions), showed that higher HIV and anaemia prevalence was found in poorer and younger groups (aged > 40 years old). In contrast, higher cardiometabolic conditions prevalence was found in richer and older groups [48]. Such multimorbidity disparity suggests that population targeted lifestyle prevention of multimorbidity in LMICs should consider both CDs and NCDs risk factors. Another study in South Africa [49], found that income was consistently associated with multimorbidity, along with risk factors such as social assistance (Odds Ratio, OR 2.35; 95% Confidence Interval, CI 1.59–3.49), residence (OR 0.65; CI 0.46–0.93), smoking (OR 0.61; CI 0.38–0.96); obesity (OR 2.33; CI 1.60–3.39), depression (OR 1.07; CI 1.02–1.11) and health facility visits (OR 5.14; CI 3.75–7.05). Addressing multimorbidity disparity based on income and welfare issues appears to be more relevant to multimorbidity prevention in LMICs. A symbiotic relationship has also been reported between NCDs, its risk factors and poverty, and due to large healthcare expenditure associated with having multimorbidity [50].

The need for promoting healthy lifestyle behaviours in preventing multimorbidity has been highlighted by others [45,51]. However, current evidence suggests that economical disparities and the double burden of CDs and NCDs are responsible for determining the contribution of lifestyle factors to multimorbidity in LMICs. Lifestyle preventative approaches could have a positive impact on reducing multimorbidity with targeted interventions considering such disparities.

**Table 1:** Multimorbidity (MM) determinants across low and middle-income countries (LMICs).

Study Type and Region	MM Prevalence	Chronic Conditions Included (Forming MM)	Determinants of MM
Cross-sectional survey among older adults, Bangladesh [26]	Overall MM prevalence (56.5%).	MM: Cumulative Illness Rating Scale (domains of disease, hypertension, ear-nose and throat, upper and lower GI, respiratory, cardiovascular, musculoskeleta)	Age: High prevalence in 60–69 y.o. (78.2% with two NCDs, 73.6% with three NCDs and 92.95 with four or more NCDs. Female sex: (prevalence of 64.2%). Hypertension: Higher MM among hypertensive (95.7%).
Systematic review of 70 community-based surveys, 31 LMICs and 18 HICs [28]	Pooled MM prevalence in LMICs (43.5%)	MM: 4 to 40 diseases with hypertension, diabetes, arthritis and stroke	*Female sex:* 21 studies showed higher MM prevalence in women than men (e.g., in South Africa 74% vs. 26%), 4 studies showing the reverse. *Age:* MM positively associate with age (e.g., 88.1% in women and 76.3% in men prevalence in > 85 y.o).
Nationwide WHO Stepwise survey for NCDs risk factors in Uganda [18,44]	Overall prevalence of Hypertension was 26.4%, was concurrent with obesity, and 2% impaired fasting glucose (1.2 diabetes). 56 · 4% had at least two risk factors (mainly high blood pressure and BMI)	MM: Hypertension, diabetes and obesity	*Age:* Hypertension was high among ≥ 50 y.o (PRR = 3.57). *BMI:* Hypertension prevalence was highest (PRR = 1.67) among obese (BMI ≥ 30 kg/m^2^). *Rurality, Sex:* Lower hypertension in rural than urban (25.8% vs. 28.2% N.S.). Higher hypertension in men vs. women (28.3% vs. 25.2% N.S.).
Review on global hypertension burden [32]	Higher overall prevalence of hypertension in LMICs (31.5%, 1.04 billion people) than in HICs (28.5%, 349 million people), and was concurrent with obesity	MM: Hypertension and obesity	*Lifestyle factors:* high sodium intake, low potassium intake, alcohol consumption, physical inactivity and unhealthy diet. *Obesity*: increases risk of hypertension. *Socioeconomic status:* increases risk of hypertension.
WHO STEPwise and GSHS surveys report in 33 countries in the WHO African Region [19]	31 surveys indicated obesity median prevalence of 11% (ranged 2% in Madagascar to 25% in Seychelles). Hypertension median prevalence of 31% (17% to 40%). Diabetes indicated (fasting blood glucose ≥ 6.1 mmol/L), prevalence was 8% (3% in Togo and Benin to 23% in Niger).	MM: Obesity, hypertension, diabetes	*Sex:* Females are three times more likely to be obese than males (median prevalence of 15% and 5% respectively). Adult males are more likely to be hypertensive than adult females (median 29%). *Lifestyle:* Tobacco use, unhealthy diet, insufficient physical activity and the harmful use of alcohol associated with higher NCDs risk.
A cross-sectional analysis of Demographic and Health Surveys in 33 countries in Sub-Saharan Africa region [33]	Hypertension was highest in women in Lesotho (17.3%) and lowest in women in Burundi (1.0%). Anemia was most prevalent in Gabon (60.6%) and obesity was most prominent in Lesotho (19.9%), Gabon (18.9%) and Ghana (15.6%).	MM: Hypertension and Obesity. Anaemia was also prevelant concurrently with those MM	*BMI:* Associated with hypertension and anaemia. *Lifestyle factors*: Smoking, fruits, vegetables and alcohol, exercise were modifiable factors associated with hypertension. *Non-modifiable factors *(Age, residence, religion, education, wealth index, marital status, employment and number of children ever born) associated with hypertension and anaemia.
A population based survey in China and Korea [42]	31% in China had MM, higher proportions in cities. 24% had MM in Korea	MM: Hypertension, diabetes, arthritis and stroke	*Rurality:* Associated with reduced risk of MM *Socioeconomic status:* disparity of MM in rural vs. urban China.
A cross-sectional survey in Southern (18,137 middle aged and elderly), China [46]	20.8% had MM. Most common MM pair was hypertension and diabetes (4.6%). Hypertension was 27.9%.	MM: Hypertension, diabetes	*Health-related Quality of Life:* Physical, mental, emotional, and social functioning.
Cross-sectional (16,000 clinic visits), Kenya [41]	12.7%and 9% (M and F) Bronchitis; 10.8% and 11.2% (M and F), acute respiratory infection; virosis 12.7% (both M and F).	MM: Acute respiratory infections, bronchitis, virosis	*Environmental conditions:* Air pollution reduction, drinking water provision, and waste management in slums can have an influence on health status. *Air pollution:* Cause and aggravating factor of respiratory diseases (chronic obstructive pulmonary disease, asthma, pharyngitis).

Table abbreviations: BMI–Body Mass Index; HICs–High Income Countries; LMICs–Low and Middle-Income Countries; MM–Multimorbidity; NCDs–Non-communicable Diseases; PRR–Prevalence Rate Ratios; GSHS–Global School Health Survey. WHO–World Health Organisation. GI–Gastrointestinal. y.o.–years old. M–Male. F–Female N.S. statistically non-significant difference. Multimorbidity is defined as having two chronic conditions or more.

## 4. Future Scope for Multimorbidity Prevention through Lifestyle Interventions in LMICs

Current data about multimorbidity prevention using lifestyle interventions are mainly based on populations in HICs, and all support a strong inverse relationship between healthy lifestyle behaviours and multimorbidity prevalence. For example, a recent prospective cohort study involving 291,778 participants from seven European countries, showed that the risk of developing multimorbidity (e.g., conditions of CVD and type-2 diabetes) is lower for individuals leading healthier lifestyle featuring increased physical activity levels, dietary habits based on Mediterranean nutritional components, reduced smoking, and lower BMI status [52]. Earlier data from the English Longitudinal Study of Ageing had also associated multimorbidity with multiple unhealthy lifestyle practices, with physical inactivity increasing multimorbidity by 33% [53]. A similar association was reported in a 10-year follow-up of Finnish population-based cohorts between 1982–2012 showing that lifestyle risk factors predict multimorbidity incidence rate [54]. Therefore, the contribution of lifestyle practices in the development of multimorbidity is well reported in HICs [55,56,57], but not in LMICs.

Global efforts by WHO attempted to control and prevent NCDs through developing a global action plan (2013–2020) [58]. The strategy included nine targets for nations to achieve by the year 2025, focusing on preventing top causes of NCDs (CVD, cancer, type-2 diabetes, chronic respiratory problem) related premature mortality by 25% [31,58]. Key targets included lifestyle interventions based on promoting reduction in salt intake, tobacco alcohol use, increasing physical activity, increasing technology, and building capacity. However, many LMICs do not have the required capacity to implement such globally-derived health promotion initiatives and suffer from disintegrated health services [2]. This suggests a need for more localised preventative approaches to address multimorbidity. More recent systematic reviews have suggested four approaches specific to LMICs including risk reduction, policy development, advocacy and strengthening of health systems for early diagnosis and treatment of NCDs [16]. WHO has also implemented a ‘best buy approaches’ intended to support governments to implement NCD interventions [58,59], including reducing lifestyle-related risk factors (tobacco use, harmful use of alcohol, unhealthy diets, and physical inactivity) for CVD, diabetes and cancer [16]. However, this “best buy” initiative has been criticised for a limited focus on health behaviour awareness rather than taking a multi-pronged intervention approach [16]. Such common “downstream” policies (e.g., raising awareness and reducing salt intake to prevent hypertension) have limited effectiveness in changing population’s behaviours [60,61]. More personalised strategies involving multi-faceted (reformulation, branding and media campaigns) and ‘upstream’ population-wide intervention-based policies are likely to be more effective [61,62].

Lifestyle intervention in LMICs is likely to be hindered by multimorbidity associated economic disparities within the same LMIC [17], where NCDs among affluent communities and households, is more related to adopting Western lifestyle and dietary habits [63]. Thus, an individualised approach using personalised lifestyle interventions (nutrition-based models, physical activity approaches), should focus on adopting an economical-based model, in which community disparities are addressed. Physical activity prevention is a known strategy for reducing NCDs and multimorbidity [52,54]. For example, adopting carefully planned personalised physical activity models, structured or unstructured, intense, or moderate exercise, can target high-risk individuals with a cluster of CVD and diabetes multimorbidity risks [64]. Furthermore, lifestyle intervention components of locally driven physical activity models and locally sourced functional foods, could also protect against pathogenic viral infections, and provide joint CDs and NCDs prevention [65]. Personalised approaches that are locally-driven are likely to reduce multimorbidity in LMICs if informed by culturally relevant nutritional, physical activity, and educational strategies as part of a multicomponent lifestyle intervention model [64,65,66].

Education and counselling are also important, given the lack of knowledge and awareness about various chronic conditions (e.g., hypertension) in women and in rural areas within LMICs [67]. Thus, it can be advocated within its specific context to promoting healthier lifestyle practices, especially consumption of local bioavailable nutrients combined with sedentary reduction and affordable physical activity engagement. Addressing multimorbidity’s comorbidities and risk factors within LMICs is another barrier to promoting lifestyle interventions for multimorbidity prevention. For example, high prevalence of epilepsy in South Africa 7.8 per 1000 people was concurrent with lower levels of physical activity than the general population due to fears that exercise could trigger seizures which predisposes these individuals to a sedentary lifestyle, poor physical fitness, and low sport participation [68,69,70]. Lifestyle behavioural interventions with such challenges require adequate multi-sectorial public health collaboration in LMICs, which in turn adds to LMICs economic burdens [70]. Understanding multimorbidity prevention barriers in LMICs require further research. Nonetheless, lifestyle interventions could form an immediate prevention strategy of multimorbidity in LMICs.

## 5. Conclusions

LMICs are experiencing a fast-paced epidemiological transition towards multimorbidity, characterized by clusters of NCDs, which require public health interventions. Multimorbidity determinants include increased age, female sex, environment, lower socio-economic status, obesity, and lifestyle behaviours, especially poor nutrition, physical inactivity. However, LMICs, especially Sub-Saharan Africa, suffer from local and regional economic disparity, which hinders health promotion initiatives. Perhaps multicomponent lifestyle interventions targeted towards highly prevalent multimorbidity clusters, especially hypertension, diabetes and cardiovascular disease could provide an early effective prevention of multimorbidity in LMICs, especially within Sub-Saharan African countries with emerging socio-economic disparity. Lifestyle behavioural approaches in Sub-Saharan African can inform public health policies for reducing an imminent multimorbidity rise within LMICs.

## Data Availability

Not applicable.

## Abbreviations

BMI	Body Mass Index
CDs	Communicable Diseases
DHS	Demographic Health Survey
HIC, HICs	High Income Countries
LMIC, LMICs	Low and Middle Income Countries
MM	Multimorbidity
NCDs	Non-communicable Diseases
CVD	Cardiovascular disease
